# Complete chloroplast genome sequence of *Camellia rhytidophylla*, comparative and phylogenetic analysis

**DOI:** 10.1080/23802359.2020.1856010

**Published:** 2021-01-16

**Authors:** Xiao-Fei Liu, Ying-Bo Sun, Li-Li Huang, Ye-Chun Xu, Chao-Yi Zhao, Bo Yu

**Affiliations:** Environmental Horticulture Institute, Guangdong Academy of Agricultural Sciences, Guangdong Key Lab of Ornamental Plant Germplasm Innovation and Utilization, Key Laboratory of Urban Agriculture in South China, Ministry of Agriculture, Guangzhou, Guangdong, China

**Keywords:** *Camellia rhytidophylla*, chloroplast genome, phylogenetic analysis

## Abstract

*Camellia rhytidophylla* is an endangered plant with economic value. Using Illumina sequencing, the chloroplast genome of *C. rhytidophylla* was sequenced and analyzed in this study. The complete chloroplast genome is 157,073 bp in length, which consisted of a pair of inverted repeat regions of 26,055 bp (IRa and IRb) separated by a large single-copy region (LSC) of 86,680 bp and a small single-copy region (SSC) of 18,283 bp. The *C. rhytidophylla* chloroplast genome encodes 135 genes, including 87 protein-coding genes, 37 tRNA genes, 8 rRNA genes, and 3 pseudogenes. Sequence comparison analysis with the chloroplast sequences of 28 other *Camellia* plants found that *C. rhytidophylla* had the closest relationship with *C. szechuanensis*. This study provides a theoretical basis for the analysis of the distant relationship of *Camellia*.

*Camellia rhytidophylla* belongs to the genus *Camellia* in the family Theaceae. *C. rhytidophylla* was planted in the Environmental Horticulture Research Institute of the Guangdong Academy of Agricultural Sciences (N23°23′, E113°23′, Guangzhou, China) (No: EHRIGAASC003). *C. rhytidophylla* can be used for landscaping. Evergreen shrubs with thick, leathery leaves and uneven leaf surface. White flowers, solitary in the axillary parietal lobe. The fruit is nearly spherical with irregular nodular protrusions on the surface.

The chloroplast genome DNA of *C. rhytidophylla* was extracted from young leaves. Firstly, DNA was broke into fragments of 300 bp length using Covaris M220 (Covaris, Woburn, MA), and then, we constructed shotgun sequencing libraries according to the TruSeq™ DNA Sample Prep Kit for Illumina. Finally, whole genome sequencing was executed using the Illumina NovaSeq platform (Illumina, USA) (Genepioneer Biotechnologies Co. Ltd, Nanjing, China). Pair-end Illumina raw reads were filtered using Trimmomatic (Bolger et al. 2014), and these reads were mapped to the chloroplast genome of the reference species (Genbank accession number: NC_024663), using Bowtie2 v2.2.4 (Langmead and Salzberg 2012) to exclude reads of nuclear and mitochondrial origins. SPAdes 3.6.1(Bankevich et al. 2012) and Sequencher 5.3.2 (Gene Codes Inc., Ann Arbor, MI, USA) were used for *de novo* assembly to reconstruct the chloroplast genomes. A ‘genome walking’ technique was used to remove gaps (Souza et al. 2019). Jellyfish v.2.2.3 (Marcais and Kingsford 2011) was used to correct misassembled contigs. CpGAVAS (Liu et al. 2012) was used for annotation of the chloroplast genomes and OGDRAW (Lohse et al. 2007) was used to draw a circular representation. The complete chloroplast genome sequence has been submitted to Genbank with the accession number of MT663343.

The complete chloroplast genome sequence of *C. rhytidophylla* is 157,073 bp in length, containing a LSC (86,680 bp) region, a SSC (18,283 bp) region, and two inverted repeat regions (IRa and IRb, each 26,055 bp). The GC content of the overall chloroplast genome, LSC, SSC, and IR regions are 37.31, 35.32, 30.61, and 42.97%, respectively. The GC content of the two IR regions is higher than those of the SSC and LSC, which is similar to *Spathiphyllum* ‘Parrish’ (Liu et al. 2019a), *Spathiphyllum cannifolium* (Liu et al. 2019b), and *Celosia cristata* (Liu et al. 2020). The chloroplast genome is made up of 135 genes in total, including 87 protein-coding genes, 37 tRNAs, 8 rRNAs, and 3 pseudogenes.

The whole genome was used for phylogenetic tree analysis. First, we use MAFF v7.427 (Katoh et al. 2005) auto mode to align each sequence. The gaps in the alignment were removed using the program trimAl with ‘-nogaps’ v 1.4 (Capella-Gutierrez et al. 2009). Finally, MrBayes v3.2.7 (Fredrik et al. 2012) was used to construct the phylogenetic tree (Figure 1). And the result suggested that *C. rhytidophylla* had the closest relationship with *C. szechuanensis*. This study will be useful for further analysis of genetic diversity, molecular markers, and molecular breeding in *Camellia*.

**Figure 1.**
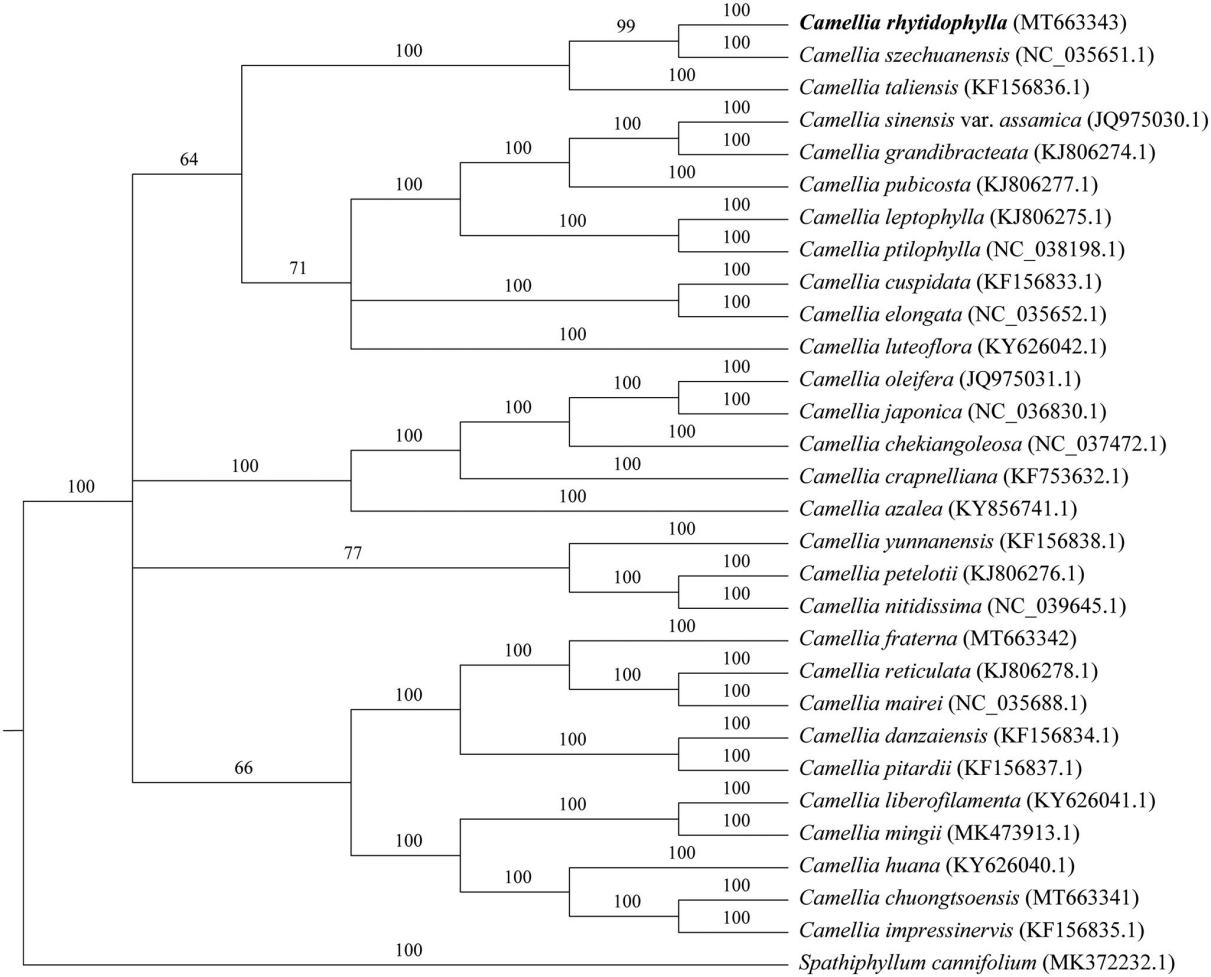
Phylogenetic tree reconstruction of 30 species based on sequences from whole chloroplast genomes. All the sequences were downloaded from NCBI Genbank.

## Data Availability

The data that newly obtained at this study are available in the NCBI under accession number of MT663343 (https://www.ncbi.nlm.nih.gov/nuccore/MT663343).
